# Use of Flow-Volume Loops on a Mechanically Ventilated Pediatric Patient as a Diagnostic Tool for Fixed Airway Obstruction

**DOI:** 10.7759/cureus.39765

**Published:** 2023-05-31

**Authors:** Lesa A Ward, Chad J Pezzano, Richa S Nathan, Ilana Harwayne-Gidansky

**Affiliations:** 1 Department of Cardiorespiratory, Albany Medical Center, Albany, USA; 2 Department of Pediatrics, Albany Medical College, Albany, USA

**Keywords:** ventilator graphics, tracheal rings, otolaryngology, vascular ring, mechanical ventilation, flow-volume loop

## Abstract

The flow-volume loop (FV-loop) provides a graphical representation of the inspiratory and expiratory flow of both mechanically provided breaths and patient-triggered breaths during invasive mechanical ventilation. The FV-loop on the ventilator-delivered breath displays the active inspiratory flow reflective of lung compliance and the passive expiratory flow reflective of airway resistance. Our case report highlights the importance of the FV-loop in determining a fixed airway obstruction. A five-month-old male presented to the emergency department with worsening respiratory distress in the setting of rhino-enterovirus. He was admitted to the pediatric intensive care unit (PICU) and intubated for acute hypoxic respiratory failure. The findings on his ventilator FV-loop graphics denoted a fixed airway obstruction, as seen by the truncation of inspiratory and expiratory flow. The patient was subsequently found to have a left pulmonary artery (LPA) sling with a vascular ring and several complete tracheal rings. He was transferred to a referral institution for operative management, returned to our PICU, and discharged home after 47 days of hospital management. During mechanical ventilation, FV-loops can be effectively utilized to assist in the diagnosis of fixed intra- or extra-thoracic airway obstructions.

## Introduction

Understanding pulmonary mechanics during mechanical ventilation is essential for safe and effective ventilatory support. With the compounding difficulty of the heterogeneity of pediatric mechanics and the severity of disease states, it is critical to evaluate the mechanical ventilator scalar waveforms and loops. Furthermore, understanding the morphology of an abnormal flow-volume loop (FV-loop) can aid in narrowing down the differential diagnosis of airway abnormalities.

A five-month-old male was admitted to the pediatric intensive care unit (PICU) for acute hypoxic respiratory failure requiring intubation and mechanical ventilation. The flow-volume loop (FV-loop) on the mechanical ventilator was suggestive of a fixed airway obstruction [1]. This finding guided the provider team to expand their differential diagnosis and facilitated a plan for the proper management of this patient. The patient was diagnosed with a left pulmonary artery (LPA) sling and several complete tracheal rings, which were initially made evident by the truncation on his FV-loop and confirmed with bronchoscopy and CT imaging [2].

## Case presentation

**Figure 1:**
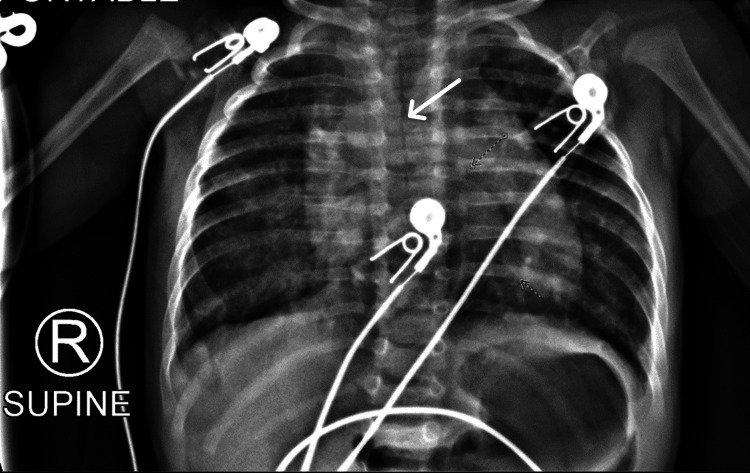
Chest radiograph. The chest radiograph before intubation is consistent with the acute respiratory viral infection. Additionally, there is a significant narrowing of the trachea noted.

A five-month-old term male with a known history of an atrial and ventricular septal defect without prior apparent clinical sequelae presented to our emergency department with acute hypoxemic respiratory failure secondary to polymerase chain reaction (PCR)-diagnosed rhino-enterovirus. He was placed on non-invasive ventilation and admitted to our PICU for further care. The chest radiograph was suggestive of viral airway disease, including bilateral perihilar bronchial thickening and interstitial infiltrates and a narrowing of the trachea (Figure 1). Additionally, there were no signs of monophasic or biphasic stridor prior to intubation. The patient underwent endotracheal intubation with a 3.5-mm endotracheal tube soon after admission to the PICU for severe respiratory distress with corresponding respiratory acidosis on venous blood gas analysis.

The patient was placed on pressure-controlled (PC) ventilation with a PC-level of 32 cm H_2_O with a volume target of 6 mL/kg, a positive end-expiratory pressure (PEEP) of 7 cm H_2_O, a set respiratory rate of 37 bpm, and FiO_2_ of 0.4, which had shown steady correction of his respiratory acidosis. The patient received sedation and neuromuscular blockades to prevent ventilator asynchrony. The mother had reported slight bleeding during suctioning of the nasopharynx in the emergency department. At this time, there were no apparent signs of trauma, bleeding, or friability of the larynx. 

**Figure 2:**
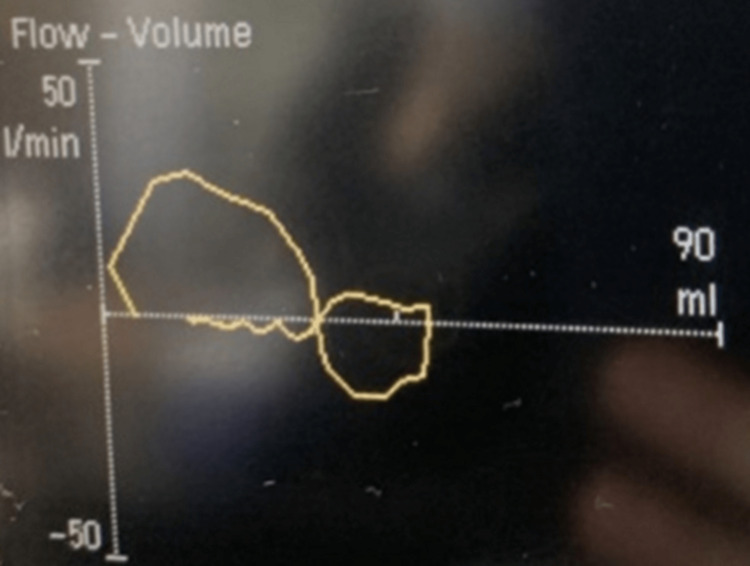
Mechanically ventilated FV-loops. The FV-loop shows a return to the x-axis on both the inspiratory and expiratory sides during mechanical ventilation. This is the site of the granulation tissue causing the majority of the fixed airway obstruction. FV: flow-volume loop.

While mechanically ventilated, the FV-loop had a “snowman-like” appearance (Figure 2). The inspiratory flow line demonstrated an abrupt return to baseline prior to the completion of the given breath. This cessation of flow is a result of a fixed obstruction, where airflow is disrupted before the breath can terminate. The airflow obstruction is disrupted at the same location on both the inspiratory and expiratory limbs. This was later determined to be the result of granulation tissue.

**Figure 3:**
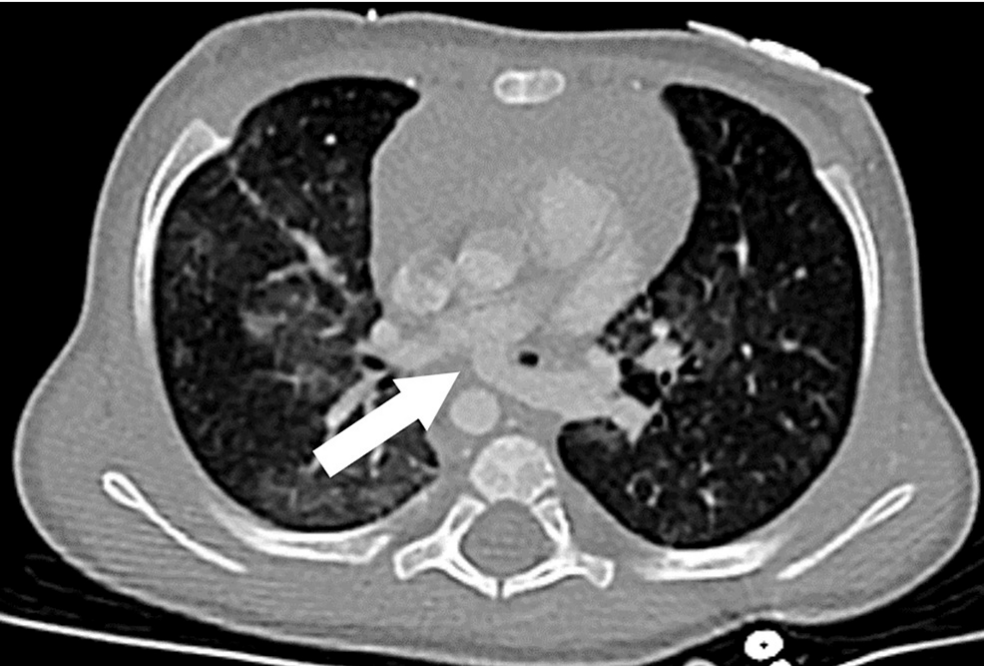
CT-imaging of LPA sling. The high-resolution chest CT imaging demonstrates the left pulmonary artery passing between the esophagus and trachea. LPA: left pulmonary artery.

**Figure 4:**
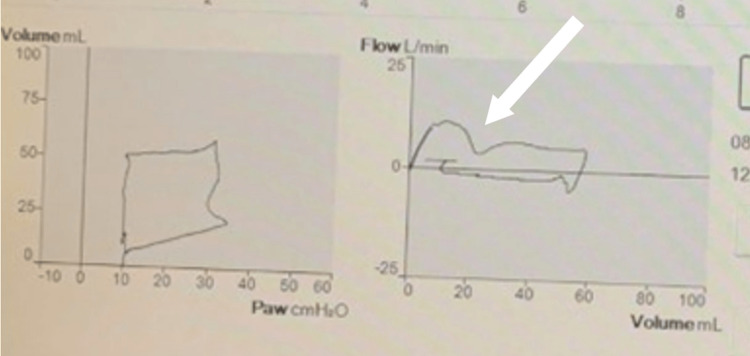
Mechanically ventilated FV-loops. The FV-loop after removal of the granulation tissue and endotracheal tube placed distal to the granulation site. FV: flow-volume loop.

The patient continued to have difficulty achieving adequate gas exchange and worsening acidemia; despite the escalation of ventilatory settings. Given the aberrant finding on the ventilator graphics and clinical decline, the patient received a chest CT with contrast (Figure 3) and was then taken to the operating room for a rigid bronchoscopy with pediatric otolaryngology. The LPA appeared to originate distally from the right pulmonary artery, which may be a substrate for a left pulmonary sling. The patient’s operative report noted severe stenosis in the mid-trachea with significant left-sided granulation. A 3.5-mm endotracheal tube was reattempted but was unable to advance with the rigid bronchoscope. The 3.5-mm endotracheal tube was chosen based on the patient's age and weight, though a 3.0-mm endotracheal tube was required to advance pass the tracheal rings. After the 2-3 cm of granulation tissue was removed, the otolaryngologist noted two complete cartilaginous tracheal rings proximal to the site of granulation, and two additional complete trachea rings distally to the granulation tissue. Severe airway stenosis was also noted distal to the granulation tissue, with complete occlusion proximal to the carina with posterior pulsatile extrinsic compression. These findings were consistent with CT imaging, which noted a posterior-pushing pulmonary artery. After the removal of the granulation tissue and placement of the endotracheal tube distal to the granulation site, the FV-loops showed improvements in flow; though, a partial disruption of flow remained on the inspiratory limb (Figure 4). The deceleration of inspiratory flow remaining represents an obstructive component, which may be a result of the complete tracheal rings and distal extrinsic tracheal narrowing.

The patient was transferred to a referral institution for operative management. He underwent a vascular ring division with diverticulum resection and reimplantation of the left subclavian artery, a division and reimplantation of the left pulmonary artery, and a slide tracheoplasty. Additionally, he underwent atrial and ventricular septal closures. The patient was extubated, stabilized, and returned to our institution for ongoing management. On return, he was weaned to room air and discharged home after 47 days of his hospitalization.

## Discussion

During mechanical ventilation, the FV-loop is a graphical representation of the breath cycle. It starts at the intersection of the flow and volume axes and moves clockwise along the x-axis as both volume and flow increase during inspiration [1]. At the end of inspiration, both volume and flow should decrease simultaneously to zero flow on the x-axis. Exhalation starts as flow and volume move towards the negative region, and the breath is considered complete when both flow and volume return to zero, assuming there is no system leak or air trapping. The mode of the ventilator determines the shape of the inspiratory flow, and the amount of flow delivered varies depending on the chosen modality [2]. For example, in true volume-control ventilation, the inspiratory flow is constant; therefore, the inspiratory portion will be square-shaped. In this case, the patient was on pressure-control ventilation, where the inspiratory flow is decelerating, thereby creating a descending flow pattern to the inspiratory flow.

While receiving mechanical ventilation, this patient required a high-pressure level to achieve adequate ventilation and a tidal volume target of 6 mL/kg. The high-pressure level was likely due to the increased airway resistance from the fixed airway obstruction. Additionally, a smaller endotracheal tube was required to bypass the obstruction, which further increased the airway resistance.

In fixed airway obstruction, the airflow is restricted due to structural changes in the airways, such as scar tissue, inflammation, or granulation. This results in a reduction of airflow during both inhalation and exhalation and may increase the difficulty of achieving adequate gas exchange. On an FV-loop, this is represented by a truncation or depression of the slope of flow. Fixed upper airway obstructions, as seen in this case, differ from variable upper airway obstructions. Fixed obstructions can be noted on the FV-loop on the inspiratory and expiratory sides, whereas a variable upper airway obstruction may change based on the pressure gradients of the distinct phases of the breath and the location of the anomaly. Normally, tracheal rings are C-shaped, which allows the trachea to expand and collapse per the patient’s airflow demand. Complete tracheal rings, like in this case, are a rare congenital disorder that inhibits the trachea’s ability to widen during inspiration. In approximately 50-79% of cases, complete tracheal rings are associated with a vascular ring, as seen in this patient [3,4].

In the current literature, FV-loops are often not listed as a part of the diagnostic modalities when approaching vascular rings, along with complete tracheal rings [5]. In a study conducted by Fulton et al. in which they investigated vascular rings, the diagnostic modalities included MRI and CT imaging, echocardiography, bronchoscopy, and barium swallow. These studies did not include FV-loops as a diagnostic measure of the subsequent upper airway obstruction that often follows a vascular ring or complete tracheal rings [6]. The benefit of FV-loops is that patient data is continuously monitored and does not require additional invasive procedures, such as bronchoscopy.

This case highlights the importance of utilizing FV-loops during mechanical ventilation in the diagnostic process for unique cases like the one described in this report. This patient’s rare conglomeration of complications, including a vascular ring, a pulmonary sling, atrial and ventricular septal defects, and concomitant tracheal rings, requires the utmost accuracy in diagnosis. The use of FV-loops in diagnostic techniques can reduce mortality related to upper airway obstructions [7].

## Conclusions

FV-loops are an important and often underutilized tool in diagnosing and locating a fixed upper airway obstruction. If evaluated, FV-loops could assist in the diagnosis of complicated airway anomalies. In fixed airway obstructions, the airflow is restricted due to structural changes in the airways. This results in a reduction of airflow during both inhalation and exhalation. This can be seen by truncation or depression in the slope of the flow.

We report an exceedingly rare clinical occurrence of a left pulmonary artery sling and several complete tracheal rings; this was found as a notable result by fixed airway obstructive FV-loops in the setting of respiratory viral illness. The mechanical ventilator FV-loops have been proven to be useful as a diagnostic tool; changes in the contour of the loop can heavily aid in the diagnosis and localization of airway obstruction such as in this case.
